# Measuring the burden of herpes zoster and post herpetic neuralgia within primary care in rural Crete, Greece

**DOI:** 10.1186/1471-2296-12-136

**Published:** 2011-12-06

**Authors:** Christos D Lionis, Constantine I Vardavas, Emmanouil K Symvoulakis, Maria G Papadakaki, Foteini S Anastasiou, Maria D Antonopoulou, Charalampos M Apostolakis, Stelios A Dimitrakopoulos, George I Fountakis, Ilias A Grammatikopoulos, John D Komninos, Dimitris K Kounalakis, Eva S Ladoukaki, Kornilia V Makri, Chrysa S Petraki, Nikos G Ploumis, Dimitra P Prokopiadou, Ioanna N Stefanaki, Nikos A Tsakountakis, Ioanna G Tsiligianni, Emmanouil N Tzortzis, Aggeliki A Vasilaki, Theodoros K Vasilopoulos, George E Vrentzos

**Affiliations:** 1 Department of Social Medicine, Clinic of Social and Family Medicine, Faculty of Medicine, University of Crete, PO Box 2208, Postal Code 71003, Greece; 2 Center for Global Tobacco Control, Department of Society, Human Development and Health, Harvard School of Public Health 677 Huntington Avenue, Boston, MA 02115, USA; 3 Primary Care Practice of Pirgos, Heraklion Postal Code 70010, Crete, Greece; 4 Health Center of Spili, Rethymnon Postal Code 74053, Crete, Greece; 5 Primary Care Practice of Mallia, Heraklion Postal Code 70007, Crete, Greece; 6 Health Center of Agia Varvara, Heraklion Postal Code 70003, Crete, Greece; 7 Primary Care Practice of Skinia, Heraklion Postal Code 70300, Crete, Greece; 8 2nd Department of Psychiatry, Aristotle University of Thessaloniki, Psychiatric Hospital of Thessaloniki, Postal Code 54124, Greece; 9 Primary Care Practice of Vraxasi, Heraklion Postal Code 72400, Crete, Greece; 10 Health Center of Anogeia, Rethymnon Postal Code 74051, Crete, Greece; 11 Primary Care Practice of Episkopi, Heraklion Postal Code 70008, Crete, Greece; 12 Primary Care Practice of Zoniana, Rethymnon Postal Code 74051, Crete, Greece; 13 Primary Care Practice of Hersonisos, Heraklion Postal Code 70014, Crete, Greece; 14 Primary Care Practice of Peza, Heraklion Postal Code 70100, Crete, Greece; 15 Primary Care Practice of Ebaros, Heraklion Postal Code 70015, Crete, Greece; 16 Primary Care Practice of Asites, Heraklion Postal Code 70013, Crete, Greece; 17 Primary Care Practice of Moxos, Heraklion Postal Code 70005, Crete, Greece

## Abstract

**Background:**

Research has indicated that general practitioners (GPs) have good clinical judgment in regards to diagnosing and managing herpes zoster (HZ) within clinical practice in a country with limited resources for primary care and general practice. The objective of the current study was to assess the burden of HZ and post herpetic neuralgia (PHN) within rural general practices in Crete, Greece.

**Methods:**

The current study took place within a rural setting in Crete, Greece during the period of November 2007 to November 2009 within the catchment area in which the Cretan Rural Practice-based Research Network is operating. In total 19 GP's from 14 health care units in rural Crete were invited to participate, covering a total turnover patient population of approximately 25, 000 subjects. For the purpose of this study an electronic record database was constructed and used as the main tool for monitoring HZ and PHN incidence. Stress related data was also collected with the use of the Short Anxiety Screening Test (SAST).

**Results:**

The crude incidence rate of HZ was 1.4/1000 patients/year throughout the entire network of health centers and satellite practices, while among satellite practices alone it was calculated at 1.3/1000 patients/year. Additionally, the standardised incidence density within satellite practices was calculated at 1.6/1000 patients/year. In regards to the stress associated with HZ and PHN, the latter were found to have lower levels of anxiety, as assessed through the SAST score (17.4 ± 3.9 vs. 21.1 ± 5.7; *p *= 0.029).

**Conclusions:**

The implementation of an electronic surveillance system was feasible so as to measure the burden of HZ and PHN within the rural general practice setting in Crete.

## Background

Herpes zoster (HZ) is a common disease; its incidence among the general population is estimated to be between 3-4 persons per 1000 person-years, with previous research indicating that the incidence is higher among the elderly, while is significantly lower among those of younger age, notably before the age of 50 [1-3]. HZ itself is due to the localized reactivation of the varicella-zoster virus within the ganglia where the virus has remained dormant, usually from childhood. This reactivation is accompanied commonly by a vesicular rash in the same dermatomal distribution of the ganglion, which is frequently in adjunct with pain. This pain may linger after the disappearance of the rash itself, in the form of HZ's most common complication, post herpetic neuralgia (PHN) [4]. PHN, is defined as the pain in the involved dermatome that is still present after one month after the rash's onset and may have a serious impact on the patients quality of life and health costs as it may last for several months to years [5-7] Social, psychological, and environmental factors can influence a patient's perception of pain and their ability to cope with pain and discomfort, and these factors should be taken into account when optimal care is the aim [8]. It has further been noted that the systematic evaluation of HZ related adverse effects on health-related quality of life is still needed due to its high incidence in the population of the elderly [5].

It has been estimated that the incidence of HZ will increase worldwide in the coming decades because of the aging of the population, and possibly as a consequence of childhood varicella vaccination [9,10]. Based on recent EUROSTAT predictions for Greece this aging of the population will translate into an increase in the percentage over 65 from 18.6% to 31.7% [11].

Despite this negative prediction, Greece is a European country in which no morbidity data for chronic or acute diseases are routinely collected within Primary Health Care. This serves as a barrier of the effective monitoring of diseases in the community as well as towards the evaluation of the distribution of disease in relation to time, place, geography and individual characteristics. This lack of monitoring systems impedes the measurement of effectiveness of both preventive and treatment efforts as well as the assessment of health care needs.

With all of those in mind, within a country with current financial restrictions and where primary health care is not well developed, we designed and undertook this study to measure the burden of HZ and PHN within primary health care settings with the initial aim to test the feasibility of a pilot surveillance system within the current Greek primary health care setting. It has been noted that few studies have evaluated the patient burden and treatment of PHN in primary care, the usual locus of chronic pain management [12].

Among the study's main objectives were to measure the incidence of HZ in geographically defined primary care areas in rural Crete and to examine the demographic, clinical and psychosocial correlates of HZ in a sample of patients who visited their local PHC services. It was hypothesized that HZ patients within primary care settings have a certain and distinguishable sociodemographic, pattern of utilization, disease profile and anxiety status that could facilitate their timely identification and effective treatment. To achieve the above, a database to monitor HZ and PHN within rural primary care in Greece was created and this paper reports the HZ and PHN incidence, the utilization of primary care services as well as the anxiety level in rural primary care in Crete, while it also discusses its implications on both health care research and health policy in this country.

## Methods

### Setting

The current study took place within a rural setting in Crete, Greece between November 2007 to November 2009. General Practitioners (GPs) affiliated within the Rural Practice-based Research Network of Crete, Greece, a research initiative of the Clinic of Social and Family Medicine of the University of Crete, were invited to participate. In total 19 GPs were involved from 3 health centers and 11 satellite practices with a total patient turn over population of approximately 25, 000 subjects. The participating GPs were informed during special training days on how to handle the patients with HZ or PHN that would visit their practice so as to offer them inclusion or not within the study. All participating patients provided written inform consent, while the study protocol was reviewed and approved by the Ethics Committee of the University Hospital of Heraklion, Crete, Greece (Protocol number: 10984-16/10/2007).

### Electronic database

An online electronic record database was constructed to monitor the burden of HZ and PHN, as routinely used within other primary care settings [13]. Through this online platform that was applied for the first time within primary care in Crete, participating physicians were able to include new cases of HZ or PHN, and provide the clinical, demographical and psychosocial information requested by study protocol. This electronic database, was password protected and through which enlisted members could enter the data accordingly. The system allowed each participating GP to view both their own, and their peers performance in the study (recruitment rate). The central research team, who had the role of the database administrator, was only allowed access to sensitive and personal data. The registry website is viewable at http://fammed.med.uoc.gr/herpes.

### Questionnaires

Two questionnaires were used to collect the necessary background information. The first aimed to collect the necessary demographic, descriptive and clinical aspects of the patients with either HZ or PHN. The clinical section covered aspects of the rash (duration, location), the pain (duration, severity) and other co-morbidities. Information was also collected in regards to the diagnosis and treatment of HZ and PHN. HZ was physician diagnosed, while PHN was physician assessed and defined as the presence of pain 30 days after the rash appeared. So as to assess the extent of pain endured due to the HZ rash or to PHN, the patients were requested to quantify their pain and discomfort on a 7-point Likert scale ranging from 1 (no pain) to 7 (severe pain). The second questionnaire that was used was the Short Anxiety Screening Test (SAST), which has been previously translated into Greek and validated within the context of a primary care setting in Crete, Greece [14]. The questionnaire measured the level of anxiety that is associated with HZ and PHN. It fulfills the criteria defined by the Diagnostic and Statistical Manual of Mental Disorders, fourth edition (DSM-IV) and consists of 10 items rated on a 4 point response scale. The cumulative score can range within 10-40, with a score of 24 regarded as the cut-off point for the diagnosis of anxiety. Further information on the validation and applications of this questionnaire is available in Grammatikopoulos et al study [14].

### Measurement of the burden of herpes zoster

During the calculation of the incidence of HZ in the population both HZ cases and PHN cases were regarded as episodes of the recent Herpes virus reactivation, and thus were combined in the subsequent estimation. The crude annual incidence rate of HZ was calculated based on the number of cases per 1000 patients/year and was calculated separately for health centers and for the participating remote practices. This separation was performed as GPs in satellite practices serve a well defined population, as is not the case among GPs serving in health centers. These crude incidence rates were calculated as follows: (number of cases × 1000)/total patient population per year. Adjusted incidence density rates were calculated taking into account the number of days that the GP was in practice during that specific year as follows: (crude incidence rate × 365)/number of days in practice. The latter was assessed only for satellite practices with one operating physician per day.

### Statistical analysis

All p-values were based on two-sided tests and a significance level lower than 0.05 was defined. Continuous variables are presented as mean ± standard deviation, while qualitative variables are depicted with the use of frequencies. Student t tests and chi-squared (χ^2^) tests were used for detecting differences between sub groups in regards to their clinical characteristics whereas intensity of pain and anxiety were analyzed as continuous variables. The statistical analysis was performed with the statistical package SPSS 17.0. (Statistical Package for Social Sciences, SPSS, Inc, Illinois, USA)

## Results

### Participants

Seventy three cases of HZ and PHN were registered. Most of the participants with HZ and/or PHN were female (56.3%, n = 36). The majority were married (56.3%, n = 36) and of lower educational status (76.6%, n = 49). The mean age of the participants was 67 ± 22.3 years. Detailed information on the participants' sociodemographic characteristics are presented in Table 1.

**Table 1:** Patients' sociodemographic characteristics (n = 64 with consent)

	n	%
**Gender**		
Men	28	43.8
Women	36	56.3
Age*	67	22.3
**Marital status**		
Married	36	56.3
Single	6	9.4
Divorced	3	4.7
Widow	19	29.7
**Educational level**		
Elementary	49	76.6
Secondary	12	18.8
Tertiary	3	4.7

* Mean, standard deviation

### Health care services' utilization

Among the study participants, 96.9% (n = 62) first visited the GP, while the other 3.2% (n = 2) reported seeking advice from the pharmacist or another source of care. The majority of the patients (62.5%, n = 40) first visited their regional primary care facilities of the public health care system, while only 6.3% (n = 4) and 7.8% (n = 8) visited a private physician or a secondary hospital first respectively.

### Burden of disease

Within the two year duration of the project 73 cases of HZ or PHN were noted, 58 of which were in the acute phase of HZ (79.4%) and 15 with PHN (20.6%) at the time of case enrollment. However, consent was obtained from 64 patients (55 with acute HZ and 9 with PHN), for which personal and descriptive data were obtained. Furthermore, the burden of disease was found to differ by the time of year; through which more cases were noted during winter (32%) and spring (32%) in comparison to summer (21%) and autumn (15%).

The crude incidence rate of HZ was 1.4/1000 patients/year throughout the entire network (health centers and satellite practices), while among satellite practices alone it was calculated at 1.3/1000 patients/year. Additionally, the adjusted incidence density was calculated at 1.6/1000 patients/year, having taken into account the number of days that the practices operated. The complete number of cases of HZ and PHN seen by the GPs as well as the crude and adjusted incidence is depicted in Table 2.

**Table 2:** Crude and adjusted HZ incidence within participating general practices in rural Crete, Greece

Primary Care Unit	Acute phase HZ	PHN	Total HZ episodes	Crude Incidence^1^	Adjusted Incidence^2^
**Satellite Practices**					

SP 1	10	3	13	7.2	7.2
SP 2	1	4	5	4.5	4.6
SP 3	16	2	18	12.9	14.6
SP 4	3	0	3	0.3	0.3
SP 5	9	0	9	5	5.1
SP 6	4	1	5	0.4	0.4
SP 7	5	0	5	1.25	1.3
SP 8	1	0	1	0.6	1.4
SP 9	0	0	0	0	0
SP 10	0	0	0	0	0
SP 11	0	0	1	0	0

**Health Centers**					

HC 1	1	2	3	1.5	3
HC 2	6	2	8	1.8	1.9
HC 3	2	1	3	1.5	2.6

**Total**	**58**	**15**	**73**	**1.4**	**1.6**

1: based on the number of cases per 1000 patients/year

2: based on the number of cases per 1000 patients/year taking into account the number of days that the GP was in practice during that specific year

### Signs of HZ and PHN

Among patients with HZ, 69.1% (38/55) reported noticing the pain before the development of the HZ rash. The mean duration between the development of pain and the HZ attributed rash was 5.2 days (range: 0-15 days), while among these patients the mean duration between the development of the rash and subsequently seeking medical advice was 3.3 days (range: 0-12 days). A significant percentage of the patients noted that their rash was located on either their abdomen (29.1% n = 16), their chest (27.3%, n = 15), or in their lumbar area (12.7%, n = 7). The patient's skull and throat were locations noted in smaller percentages, at 7.3% (n = 4) and 9.1% (n = 5) respectively, while 49.1% (n = 27) of the patients were found to have a rash which covered other or overlapping anatomical areas of the body.

Among the characteristics attributed to the pain by the patients, 60% (n = 33) of patients with HZ reported the pain to cause a burning sensation, and 38.2% (n = 21) reported that it was continuous throughout the day. On the other hand, among the patients diagnosed with PHN, only 33.3% (n = 3) reported it as a burning sensation while 55.6% (n = 5) reported the pain to be continuous and 11.1% (n = 1) reported it as a painful itching sensation.

For patients with HZ, and as assessed at the time of arrival at the GP's practice and on the 0-7 Likert scale, 25.5% (n = 14) rated it as a 5/7, 23.6% (n = 13) as a 6/7 and 20.0% (n = 11) as a 4/7 on the Likert scale. Among those with PHN, 4 out of the 9 patients rated the pain with a 4/7 rating, 2 out of 9 reported a 7/7 rating, and 1 patient reported their pain to be 6/7 on the Likert scale.

Of the 64 patients with HZ and PHN, co-morbidities, such as hypertension (49.2%, n = 31), hyperlipidaimia (38.1%, n = 24), diabetes mellitus (19.0%, n = 12), coronary heart disease (17.5%, n = 11), chronic obstructive pulmonary disease (6.5%, n = 4), and malignant neoplasms (1.6%, n = 1) were noted.

### Anxiety assessment

Among the patients that were registered in the study, 35.9% (n = 23) were identified as positive in the SAST test (with a score ≥ 24), while an additional 5 patients manifested a marginal result (with a score of 22-23). When comparing the patients with HZ to the patients with PHN, the latter were found to have lower levels of anxiety, with a mean score of 17.4 ± 3.9 vs. 21.1 ± 5.7 indicating that those within the acute phase of HZ had higher SAST scores and this noted difference was statistically significant (*p *= 0.029). Moreover the self reported level of pain and the SAST score per individual were not correlated (rho = 0.204, p > 0.05). An additional analysis was performed so as to assess whether the patients level of anxiety played a role in seeking medical help from the regional GP or elsewhere, to which no differences were noted between the two groups (p > 0.05) (Mean score 20.9 ± 5.6 vs. 20.2 ± 5.6 for those that visited first their PHC physician or another physician respectively).

## Discussion

### Main findings

Most of the episodes of varicella virus reactivation in rural Crete were documented in the acute phase. The crude incidence rate calculated throughout the entire primary care network was similar to the lower end of incidence of HZ studies in community settings, which have reported annual incidence rates ranging from 1.3 to 4.8 per 1, 000 persons [15-18] without taking age into account, while among elder patients an annual incidence of 3.6 to 14.2 per 1000 has been reported [15]. Notably, this is observed despite the fact that access to the Greek national health care system is not based on a primary care gate-keeping regiment and therefore patients may seek healthcare elsewhere. Additionally, there are no existing primary care oriented data in Greece regarding the incidence of HZ and PHN, and the absence of an electronic encounter recording system within PHC could be partially responsible for the lack of such information [19]. This study provides some evidence on the feasibility of implementing a monitoring system within regional health care facilities, through the mobilization of GPs and with the provision of limited funding. With this reasoning, the region of Crete could be representative at a national level for both the estimation of the burden of HZ and PHN but also for the applicability of an electronic surveillance system for disease monitoring within PHC in Greece. To our knowledge, it is the first effort in reporting the frequency of HZ in primary care in Greece, an effort well established in UK and the Netherlands years ago [20].

### HZ patient profile

This study revealed that the common HZ profile in rural Crete were lower educated, elder females with participants in the acute phase of HZ reactivation found to have higher levels of anxiety when compared to participants with PHN. Indeed, previous research has indicated that those living alone, and showing increased anxiety or depression are at high risk of PHN persisting [21,22]. Assuming the effects of these psychosocial variables are causal, it has been hypothesized that PHN would be reduced by interventions that either encourage patients' participation in daily activities to the greatest extent possible or reduce patients' emotional distress [23]. Within the context of our study, and as assessed with the use of the SAST questionnaire, participants during the acute phase of HZ were found to have a higher anxiety score, a fact which we most likely attribute to their personal response to the intense combination of discomfort and pain through the development of the rash/pain.

### Implications

Research on the cost effectiveness of herpes zoster vaccination among the elderly in the US has indicated that the provision of 1 million vaccines would lead to a US$ 82-103 million reduction in health care costs due to the diagnosis, treatment and avoided complications [24]. In the US, the direct medical cost burden of HZ in the US is high, exceeding $US1000 per HZ patient. This direct medical cost may be nearly twice as high in immunocompromised patients and four times as high in the subset of HZ cases with PHN [25]. There are studies, which report a lower incidence as well as less severity of HZ among individuals vaccinated with varicella vaccine [26,27]. Investigation and detailed monitoring of the effect of vaccination against varicella into the overall HZ incidence, represent special research targets and could foster research within primary care [19].

In addition to the above, a significant percentage of the study population had other co-morbidities including diabetes, renal failure, malignancies and coronary heart disease a fact that would further reduce patient quality of life and health care expenditure. As indicated by other researchers, the healthcare costs associated with PHN are substantially greater than those associated with herpes zoster pain that is resolved within 30 days [28,29]. It is this above information regarding the costs associated with prolonged pain that would be especially relevant in determining the cost-effectiveness of emerging prevention strategies [30]. The role of the GP in the above strategy is significant, as research has indicated that family physicians have good clinical judgment in regards to diagnosing herpes zoster. A clinical study by Opstelten et al., corroborated the above fact within a primary care setting, and found that the clinical diagnosis could be confirmed serologically in 91% of patients with signs and symptoms of herpes zoster [31].

### Strengths and weaknesses

This observational study has several limitations. Combining cases of HZ and cases of PHN may overestimate the incidence of HZ. The small number of patients may limit the ability to detect potentially significant associations and this factor may have been introduced by a lacking or deficient electronic patient records system that impedes the disease's monitoring. A selection bias may be introduced since the study included patients who were seeking medical care at the participating primary care centers. It is possible that some individuals developing very mild cases of HZ may not have sought medical care. Recall bias may have also affected the study results since some information was collected by self-report. Moreover we must state that the health care system in Greece, is not based on a gate-keeping system and therefore it is possible that some patients with HZ or PHN may have visited GP's or other physicians that serve either public or private sector outside of the area under their responsibility. It can be lead to an underestimation of both the incidence of HZ and the burden of this disease. Another point of concern is the generalisability of the results to the entire population of Greece, mostly in regards to the participants with PHN. It is also possible that the patients with less severe pain did not consult a GP. However, this current study is the first to provide information on epidemiological aspects of HZ in Greece as also provide an insight into the use of the primary care services based on an electronic database. Finally, due to its cross-sectional design, no causality can be attributed from these findings in regards to the association between patient's increased anxiety or depression and the occurrence of PHN, a fact that not even prospective studies have indicated to date [20]. However, it was clear that the main contribution of this study was the measurement of the burden of the HZ that the selected primary care services in rural Crete are invite to manage.

## Conclusions

The study provides evidence on the feasibility of primary care surveillance systems to monitor acute or chronic disorders within Greece a fact previously noted in northern European settings [32]. Such initiatives seem very promising for HZ morbidity reporting in a country with limited resources that has a limited use of electronic databases. The current study provides also some evidence for the timely identification of HZ patients within primary care settings. Patients with a higher risk of developing HZ that are identified, might benefit from future preventive strategies. Moreover, the knowledge of a HZ profile could assist physicians in better understanding the disease characteristics and could further facilitate timely detection within the busy schedule of primary care physicians.

## List of Abbreviations

HZ: Herpes Zoster; PHN: Post Herpetic Neuralgia; GP: General Practitioner; SAST: Short Anxiety Screening Test; PCP: primary care practice.

## Competing interests

The Clinic of Social and Family Medicine at the School of Medicine, University of Crete, under the scientific coordination of CL, has received a grant by the Vaccine Business Unit (Sanofi Pasteur MSD) of VIANEX S.A. The established standards were followed with the University of Crete's Special Account for Research managing the funding. The organization funding this study had no role in the design and conduct of the study; the collection, analysis, and interpretation of the data; or the preparation, review or approval of the manuscript.

## Authors' contributions

CL had the main role in study conception and design. CV, EKS contributed to study protocol preparation, while author CL, EKS, CV and MP contributed to manuscript preparation and data interpretation. All the remaining authors contributed to data collection and management. All authors have read and approved the final manuscript.

## Pre-publication history

The pre-publication history for this paper can be accessed here:

http://www.biomedcentral.com/1471-2296/12/136/prepub
